# The *de novo* and salvage pathways of GDP-mannose biosynthesis are both sufficient for the growth of bloodstream-form *Trypanosoma brucei*

**DOI:** 10.1111/j.1365-2958.2012.08026.x

**Published:** 2012-04

**Authors:** Sabine Kuettel, Majken C T Wadum, Maria Lucia S Güther, Karina Mariño, Carolin Riemer, Michael A J Ferguson

**Affiliations:** 1 Division of Biological Chemistry and Drug Discovery, College of Life Sciences, University of DundeeDundee DD1 5EH, Scotland, UK

## Abstract

The sugar nucleotide GDP-mannose is essential for *Trypanosoma brucei*. Phosphomannose isomerase occupies a key position on the *de novo* pathway to GDP-mannose from glucose, just before intersection with the salvage pathway from free mannose. We identified the parasite phosphomannose isomerase gene, confirmed that it encodes phosphomannose isomerase activity and localized the endogenous enzyme to the glycosome. We also created a bloodstream-form conditional null mutant of phosphomannose isomerase to assess the relative roles of the *de novo* and salvage pathways of GDP-mannose biosynthesis. Phosphomannose isomerase was found to be essential for parasite growth. However, supplementation of the medium with low concentrations of mannose, including that found in human plasma, relieved this dependence. Therefore, we do not consider phosphomannose isomerase to be a viable drug target. We further established culture conditions where we can control glucose and mannose concentrations and perform steady-state [U-^13^C]-d-glucose labelling. Analysis of the isotopic sugar composition of the parasites variant surface glycoprotein synthesized in cells incubated in 5 mM [U-^13^C]-d-glucose in the presence and absence of unlabelled mannose showed that, under physiological conditions, about 80% of GDP-mannose synthesis comes from the *de novo* pathway and 20% from the salvage pathway.

## Introduction

The African trypanosomes are protozoan parasites that divide in the blood of the mammalian host and cause Human Sleeping Sickness and Nagana in cattle.

In the mammalian bloodstream form of the parasite, a dense coat of 5 × 10^6^ GPI-anchored variant surface glycoprotein (VSG) homodimers protects the plasma membrane (Mehlert *et al*., 1998). In addition to VSG, the parasite expresses a number of less abundant glycoproteins some of which are specific to, and essential for, the infectious bloodstream form of the parasite. Examples include the ESAG6/ESAG7 heterodimeric transferrin receptor (Steverding, 2000), the major lysosomal glycoprotein p67 (Alexander *et al*., 2002; Peck *et al*., 2008) and the membrane-bound histidine acid phosphatase TbMBAP1 (Engstler *et al*., 2005). All of these molecules contain d-mannose (Man), d-galactose (Gal) and d-*N*-acetylglucosamine (GlcNAc), with Man present in the Man_3_GlcNAc_2_ cores of all glycoprotein N-linked glycans, in the outer sugars of the Man_5_GlcNAc_2_ to Man_9_GlcNAc_2_ oligomannose N-linked glycans and in the Man_3_GlcN-*myo*-inositol cores of all protein-linked and free GPI structures.

**Figure 1:**
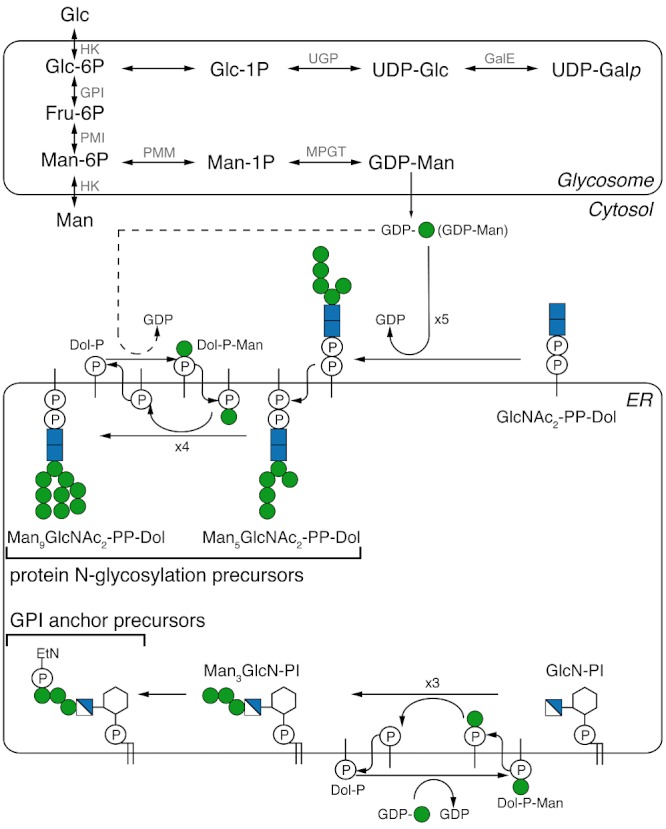
Mannose metabolism in *Trypanosoma brucei*. The parasite is able to take up Glc and Man from the environment and transport them into the glycosome. The *de novo* pathway to GDP-Man involves the conversion of Glc to Glc-6P to Fru-6P to Man-6P to Man-1P to GDP-Man. The salvage pathway involves the conversion Man to Man-6P to Man-1P to GDP-Man. The *de novo* pathway from Glc to UDP-Gal*_p_*, via UDP-Glc, is also shown. The figure also shows the fate of GDP-Man as the direct donor for the synthesis of Man_5_GlcNAc_2_-PP-Dol and as the indirect donor (via Dol-P-Man) to Man_9_GlcNAc_2_-PP-Dol Man. These molecules are used in the endoplasmic reticulum (ER) of the parasite as donors for the oligosaccharyltransferases of protein N-glycosylation. GDP-Man is also used as the indirect donor (via Dol-P-Man) for the synthesis of the ethanolamine-P-Man_3_GlcN-PI GPI anchor precursor. The enzyme abbreviations are: HK, Hexokinase; GPI, Glc-6P isomerase; PMI, Phosphomannose isomerase; PMM, Phosphomannomutase; MPGT, GDP-Man pyrophosphorylase; UGP, UDP-Glc pyrophosphorylase; GalE, UDP-Glc 4′-epimerase.

Eukaryotic mannosylation reactions require either the sugar nucleotide GDP-mannose (GDP-Man) or its product dolichol-phospho-mannose (Dol-P-Man). Sugar nucleotides may be formed by a *de novo* pathway, involving the bioconversion of another sugar or sugar nucleotide, or by salvage pathways, which entails the conversion of the sugar itself following its uptake by the cell and/or liberation from macromolecules by intracellular catabolism. The sequence of reactions in the eukaryotic *de novo* and salvage pathways to GDP-Man are shown in Fig. 1, along with the ultimate fates of GDP-Man and Dol-P-Man in *Trypanosoma brucei*.

Several sugar nucleotides have been shown to be essential for the survival and for infectivity of trypanosomatid parasites. Thus, UDP-Gal is essential in both bloodstream- and procyclic-form *T. brucei* (Roper *et al*., 2002; 2005; Urbaniak *et al*., 2006) and also appears to be essential in *T. cruzi* (MacRae *et al*., 2006); GDP-Man is essential for infectivity in *Leishmania mexicana* and in the growth of bloodstream-form *T. brucei* (Davis *et al*., 2004; Denton *et al*., 2010); GDP-Fuc is essential in both bloodstream- and procyclic-form *T. brucei* (Turnock *et al*., 2007) and UDP-GlcNAc is essential for the growth of bloodstream-form *T. brucei* and promastigote form *L. major* (Naderer *et al*., 2008; Stokes *et al*., 2008).

Since phosphomannose isomerase (PMI) holds a key position in the *de novo* pathway to GDP-Man, we decided to characterize the *T. brucei* enzyme (TbPMI) and to create a *TbPMI* conditional null mutant to test its essentiality and assess the relative roles of the *de novo* and salvage pathways to GDP-Man.

## Results and discussion

### Cloning, expression and characterization of TbPMI

A putative *TbPMI* gene (gene number Tb11.01.6410) was identified by blastp search (Turnock and Ferguson, 2007). The alignment of the TbPMI predicted protein sequence with those of other eukaryotes is shown in *Supporting information* (Fig. S1). According to RT-PCR, *TbPMI* is transcribed in both the procyclic- and bloodstream-form life cycle stages of the parasite (Fig. S2). Additionally, both the TriTrypDB genome sequence and Southern blotting with a *TbPMI* ORF probe (Fig. 5B) suggest that the gene is present as a single-copy gene per haploid genome.

**Figure 2:**
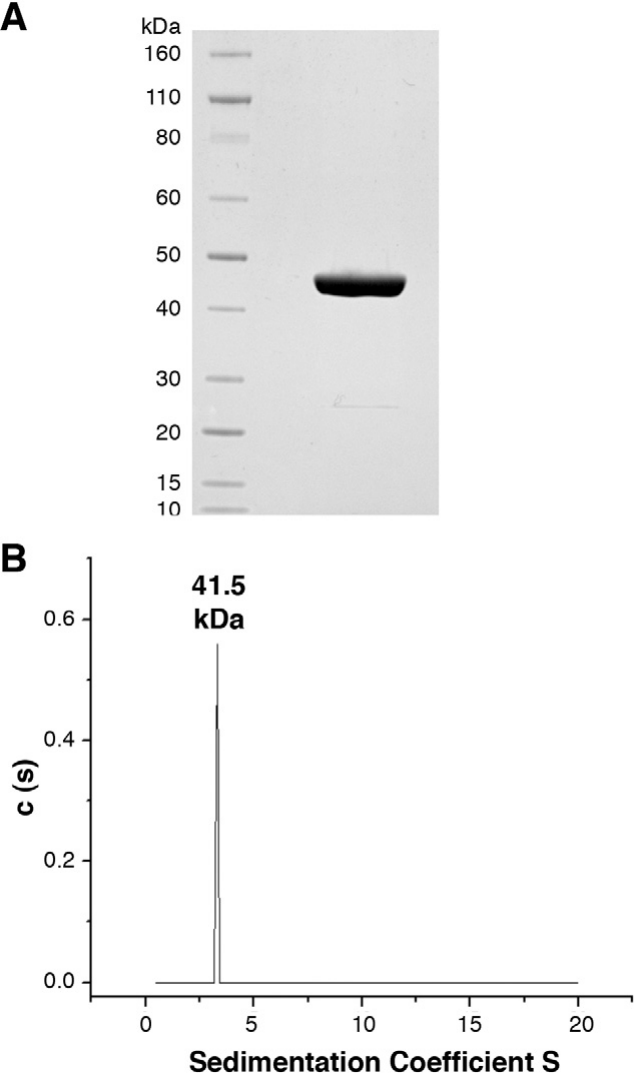
Expression and characterization of recombinant TbPMI. A. Coomassie blue-stained SDS-PAGE gel of purified recombinant TbPMI following removal of the GST-tag. B. Analytical ultracentrifugation of recombinant TbPMI following removal of the GST-tag at different concentrations [0.75 mg ml^−1^ (data shown), 0.5 mg ml^−1^ and 0.25 mg ml^−1^] suggests the enzyme is monomeric with a predicted mass of 41.5 kDa. The measured mass by MALDI-TOF mass spectrometry was 46.75 kDa.

**Figure 3:**
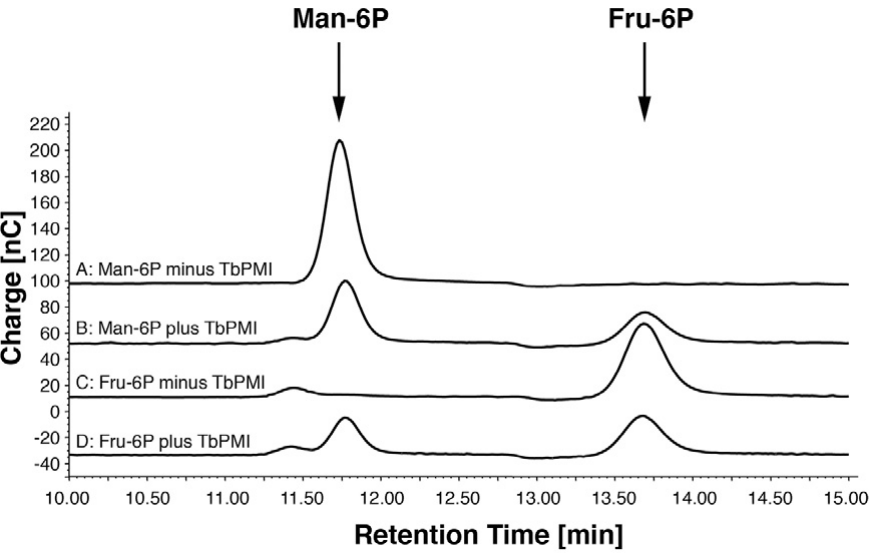
Recombinant TbPMI is enzymatically active and interconverts Man-6P and Fru-6P. High-pH anion exchange chromatography with pulsed amperiometric detection was used to detect Man-6P and Fru-6P. (A) Man-6P incubated without TbPMI. (B) Man-6P incubated with TbPMI. (C) Fru-6P incubated without TbPMI. (D) Fru-6P incubated with TbPMI.

For biochemical characterization of TbPMI, the recombinant protein was expressed with a cleavable *N*-terminal glutathione *S*-transferase (GST) tag in *Escherichia coli*, with a yield of 6 mg l^−1^ culture (Fig. 2A). The DNA sequence analysis of the TbPMI ORF cloned in this study from *T. brucei* lab strain 427 (variant 221) revealed three silent mutations (bp 219, 222 and 525) compared with the genome database sequence of *T. brucei* strain 927. Analytical ultracentrifugation of the tag-free recombinant protein showed that it is monomeric (Fig. 2B). The enzyme was found to have a pH optimum between pH 7 and 9 (Fig. S3) and to be able to convert Man-6P to Fru-6P and Fru-6P to Man-6P, as expected and as judged by high-pH anion exchange chromatography (HPAEC) (Fig. 3). The following compounds were not substrates for the enzyme: Gal*_p_*-6P, Glc-6P, GlcN-6P and GlcNAc-6P (data not shown).

### TbPMI is a glycosomal enzyme

**Figure 4:**
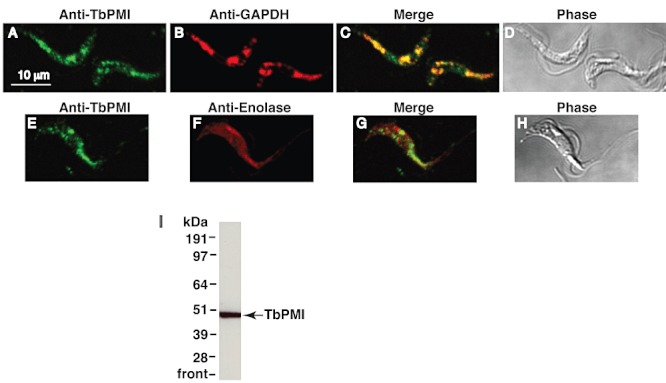
Subcellular localization of TbPMI by immunofluorescence. A–H. Paraformaldehyde-fixed bloodstream-form *T. brucei* cells stained with mouse anti-TbPMI (red) combined with rabbit anti-GAPDH (green) (A–D) or with rabbit anti-enolase (green) (E–H). GAPDH was used as glycosomal marker and enolase as cytosolic marker. Panel (C) is the merged image of (A) and (B), and (D) is the corresponding phase-contrast image. Panel (G) is the merged image of (E) and (F), and (H) is the corresponding phase-contrast image. I. A Western blot of total bloodstream-form *T. brucei* lysate developed with mouse anti-TbPMI and anti-mouse HRP.

Mouse anti-TbPMI antibody was used together with rabbit anti-GAPDH antibody as a glycosomal marker in immunofluorescence imaging. The secondary antibodies were anti-mouse Alexa 488 (green) and anti-rabbit Alexa 594 (red) respectively. The anti-TbPMI antibodies showed punctate staining that colocalized with anti-GAPDH staining, indicating that TbPMI is located predominantly in glycosome microbodies in bloodstream-form *T. brucei* (Fig. 4A–D). A similar experiment was performed with rabbit anti-enolase antibodies as a cytosolic marker (Fig. 4E–H), where no significant colocalization was detected. The anti-TbPMI antibodies were shown to be monospecific by Western blotting using a total SDS lysate of bloodstream-form *T. brucei* (Fig. 4I). The localization of TbPMI to the glycosomes is in agreement with the presence of a C-terminal peroxisome-targeting sequence type-1 (PTS1) tripeptide (SHM) and with the bloodstream- and procyclic-form glycosomal proteomes reported in Colasante *et al*. (2006). There are no published data on the localization of PMIs in *T. cruzi* or the leishmania but the former also contains a PTS1 sequence (AHI or AHM), whereas the latter contain neither PTS1 nor PTS2 sequences (Opperdoes and Szikora, 2006).

### *TbPMI* is essential to bloodstream-form *T. brucei*

**Figure 5:**
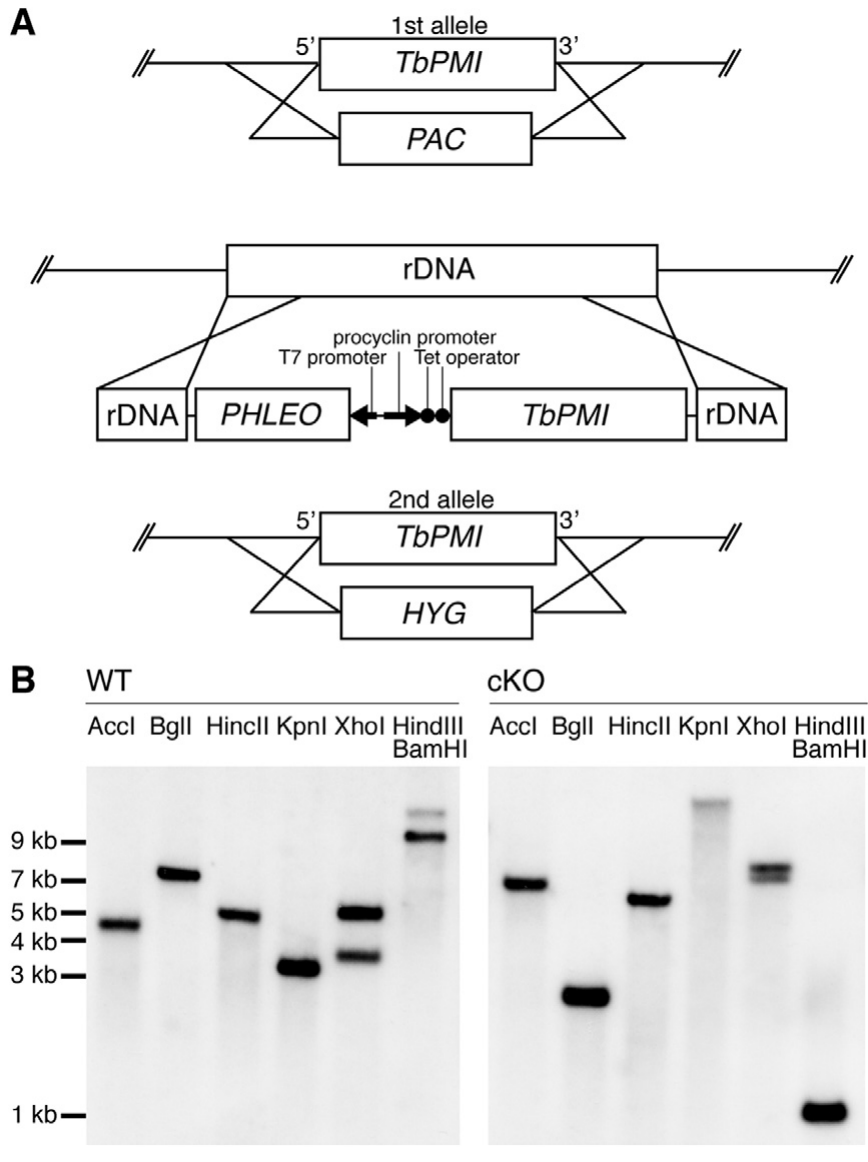
Creation of the bloodstream-form *T. brucei TbPMI* conditional null mutant. A. Schematic representation of the following genetic modifications: Replacement of the first endogenous *TbPMI* allele with *PAC* (Δ*TbPMI*::*PAC*), introduction of an ectopic tetracycline-inducible copy of *TbPMI* into the rDNA locus (Δ*TbPMI*::*PAC*/*TbPMI*^Ti^) using a pLEW100-*TbPMI* vector, and replacement of the second endogenous *TbPMI* allele with *HYG* (Δ*TbPMI*::*PAC*/*TbPMI*^Ti^/Δ*TbPMI*::*HYG*). B. Southern blots of the wild type (WT) and the *TbPMI* conditional null mutant (cKO). Aliquots of genomic DNA were digested with AccI, BglI, HincII, KpnI, XhoI, and a double digest with HindIII and BamHI, as indicated, and the resulting DNA fragments were blotted with a *TbPMI* ORF probe. The bands in the WT Southern indicate that *TbPMI* is a single-copy gene with the fragment sizes close to those predicted from the TriTrypDB genome sequence (AccI, 4170 bp; BglI, 6846 bp; HincII, 4769 bp; KpnI, 3175 bp; XhoI, 3533 bp and 4976 bp and HindIII/BamHI, 9000 bp). As expected, the bands of the cKO show a completely different pattern, demonstrating the elimination of the two endogenous gene copies and the introduction of an ectopic copy of *TbPMI* in a different locus. The double digest with HindIII/BamHI liberates the ectopic *TbPMI* gene in the cKO with the expected size of 1230 bp.

The construction of a *TbPMI* conditional null mutant is summarized in Fig. 5A. The mutant was created using a *T. brucei* bloodstream-form cell line that stably expresses the tetracycline repressor protein (Wirtz *et al*., 1999). The first *TbPMI* allele was replaced by homologous recombination following electroporation of the parasites in the presence of linear DNA containing a puromycin acetyltransferase gene *(PAC)* flanked by about 500 bp of *TbPMI* 5′- and 3′-UTR. Following selection with puromycin, a Δ*TbPMI*::*PAC* clone was selected and transformed with an ectopic, tetracycline-inducible, copy of *TbPMI*, introduced into the ribosomal DNA locus under phleomycin selection. After tetracycline induction, the second endogenous allele was replaced by a hygromycin phosphotransferase gene (*HYG*) to yield the desired Δ*TbPMI*::*PAC*/*TbPMI^Ti^*/Δ*TbPMI*::*HYG* clone. After each round of transformation, selected clones were analysed by Southern blot using a *TbPMI* ORF probe. The blot obtained after the last transfection (Fig. 5B) shows the successful introduction of the ectopic copy and replacement of both endogenous alleles in the clone used for further studies.

**Figure 6:**
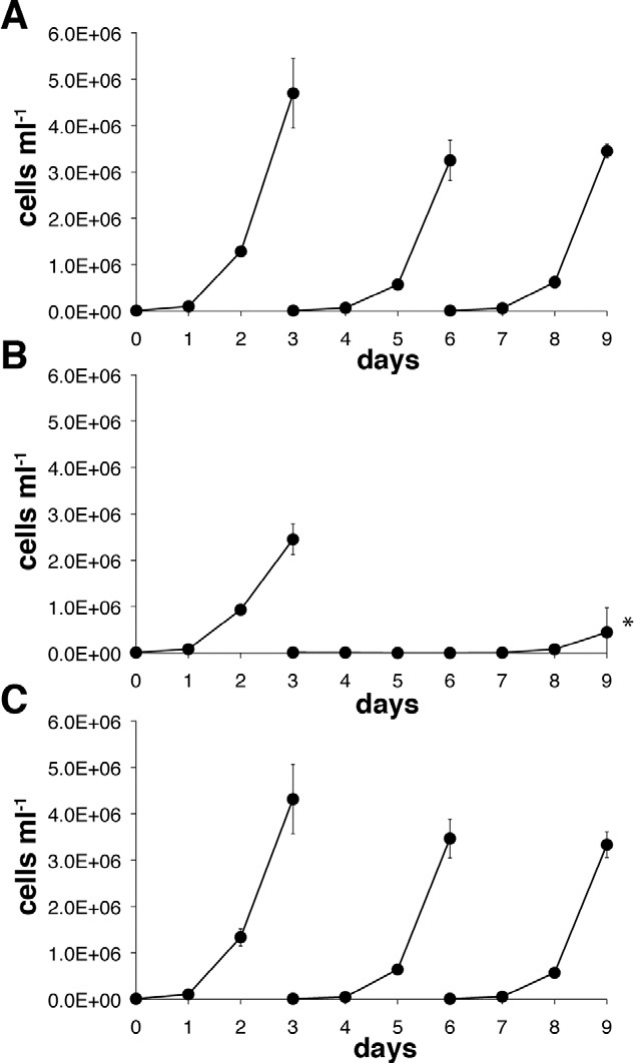
The growth of bloodstream-form *T. brucei* can be supported by either TbPMI expression or exogenous mannose. The *TbPMI* conditional null mutant was cultured for 9 days under either (A) permissive (plus tetracycline) conditions, with subculturing on days 3 and 6 or (B) non-permissive (minus tetracycline) conditions, with subculturing on day 3 only or (C) under non-permissive (minus tetracycline) conditions but with a 100 µM Man supplement, with subculturing on days 3 and 6.

Triplicate cultures of wild-type and *TbPMI* conditional null mutant cells were inoculated at 1 × 10^5^ cells ml^−1^ under permissive and non-permissive conditions (i.e. with and without tetracycline respectively) and under non-permissive conditions but supplemented with 100 µM Man. The *TbPMI* conditional null mutant cultures under permissive conditions had similar growth rates to those of wild-type cells (Fig. 6A). Under non-permissive conditions, the cells grew slowly for 3 days and on subculturing failed to grow for 5 days (Fig. 6B). Nevertheless, these cultures resumed growth on day 8. This phenomenon, where conditional null mutants become tetracycline-independent, is well known in *T. brucei* and is synonymous with gene essentiality (Roper *et al*., 2002; Marino *et al*., 2011) and references therein. The growth deficiency seen under non-permissive conditions was reversed by the addition of 100 µM Man (Fig. 6C).

This phenotype is slightly different from those reported for the *L. mexicana* promastigote PMI and GDP-Man pyrophosphorylase null mutants (Garami and Ilg, 2001a,b). In those cases, the null mutant promastigotes showed only impaired growth in culture that, for PMI but not GDP-Man pyrophosphorylase, could be partially or fully relieved when the medium was supplemented with 20 µM or 200 µM Man respectively. The *L. mexicana* pyrophosphoylase mutant was unable to infect macrophages (Garami and Ilg, 2001a), whereas the PMI mutant was infectious, though highly attenuated (Garami and Ilg, 2001b). Presumably, the presence of traces of free mannose and/or mannose derived from host glycoprotein degradation is able to support a basal level of mannose metabolism necessary for macrophage invasion and replication as the amastigote form.

### The *TbPMI* mutant can be rescued by a range of mannose concentrations in the medium

**Figure 7:**
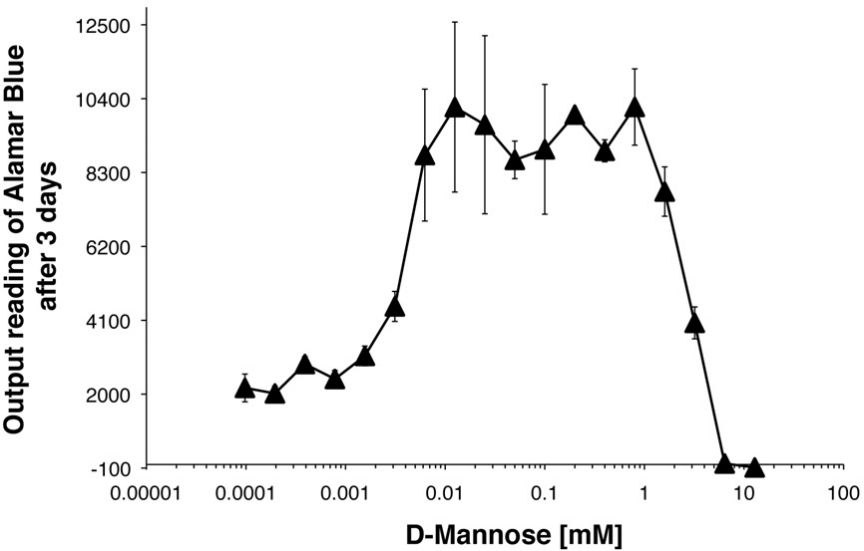
Mannose dependence of the *T. brucei TbPMI* conditional null mutant under non-permissive conditions. The growth of the *TbPMI* conditional null mutant under non-permissive conditions but with the medium supplemented with a range of free Man concentrations was assessed using 3-day triplicate cultures and the Alamar blue assay. Concentrations of free Man above 6 µM and below 1 mM supported growth equally well.

It is known that *T. brucei* can take up [^3^H]Man from the medium and incorporate it into its GPI anchor and *N*-glycan precursors and into its mature glycoproteins (Strickler and Patton, 1980; Menon *et al*., 1988; Low *et al*., 1991; Masterson and Ferguson, 1991). This suggests that GDP-Man can be synthesized by a salvage pathway, most likely involving Man to Man-6P conversion by hexokinase (see Fig. 1), and is consistent with the ability of free 100 µM Man to rescue the growth of *TbPMI* conditional null mutant under non-permissive conditions. We therefore investigated the amount of free Man in the medium necessary to rescue the *TbPMI* conditional null mutant under non-permissive conditions. Triplicate cultures were supplemented with a range of Man concentrations and the growth of the cultures assessed after 3 days (Fig. 7). Some rescue of growth could be seen with Man concentrations as low as 6 µM and the growth rates of cultures supplemented with between 6 µM and 1 mM Man were statistically indistinguishable from each other and from wild-type growth rates. We may therefore conclude (i) that under non-permissive conditions the *TbPMI* mutant is an auxotroph for Man, (ii) that a salvage pathway for GDP-Man synthesis does indeed exist in bloodstream-form *T. brucei* and (iii) that the Man → GDP-Man salvage pathway alone can fully support bloodstream-form *T. brucei* growth in culture. Interestingly, Man becomes toxic for the parasite cultures at concentrations above 2 mM, perhaps by inhibiting glycolysis (by competing for Glc uptake and/or for hexokinase activity) and/or by depleting glycosomal ATP levels by conversion to Man-6P via hexokinase and/or through the accumulation of the potentially toxic Man-6P metabolite. Similar mannose toxicity has also been noted in *L. mexicana* promastigote PMI null mutants, which have elevated hexokinase levels, but not in wild-type cells (Garami and Ilg, 2001b).

### Both the *de novo* and the salvage pathways contribute to GDP-Man synthesis in *T. brucei* under physiological conditions

**Figure 8:**
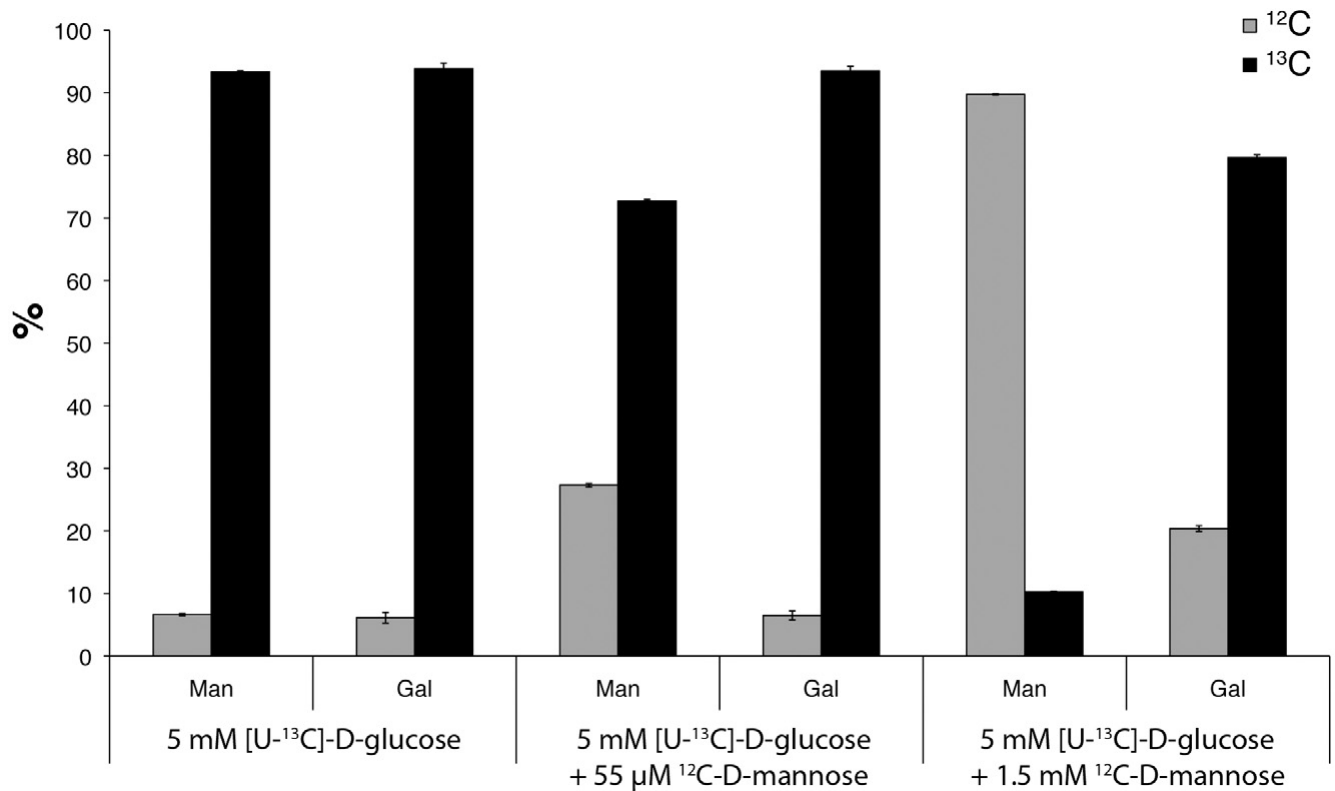
Isotopic analysis of the *de novo* and salvage pathway contributions to mannose metabolism. Samples of sVSG from cells grown in 5 mM [U-^13^C]-d-Glc under different conditions were subjected to acid hydrolysis and their Man and Gal contents were analysed as TMS-derivatives by GC-MS. The relative percentages of the ^12^C- and ^13^C-variants of Man and Gal were determined from their electron impact spectra and the results of triplicate analyses (mean ± standard deviation) are shown. In the absence of any additions, about 95% of the Man and Gal was shown to be ^13^C-labelled. However, in the presence of 55 µM Man, the proportion of ^13^C-labelled Man reduced to 75% while Gal was unaffected. In the presence of 1.5 mM Man, the proportion of ^13^C-labelled Man was further reduced to 10% and the proportion of ^13^C-labelled Gal was reduced to 80%.

While it was clear from the characterization of the *TbPMI* conditional null mutant that the *de novo* and the salvage pathways to GDP-Man can compensate for each other experimentally, it was not clear what the relative contributions of each pathway might be under physiological conditions. To address this, we first established a Glc- and Man-free culture medium, using dialysed FCS and dialysed Serum-Plus^TM^, that could support parasite growth when supplemented with a physiologically relevant (human plasma) concentrations of Glc (i.e. 5 mM Glc, rather than 25 mM Glc which is the composition of regular HMI-9T medium). We then used this medium supplemented with 5 mM [U-^13^C]-d-Glc to grow trypanosomes with and without a physiologically relevant (human plasma) concentration of Man (i.e. 55 µM) (Alton *et al*., 1997) and with 1.5 mM Man, which is close to the highest concentration of Man that the parasites can tolerate before displaying growth suppression or cell death. After 2 days (seven generations) of growth at 37°C to allow steady-state labelling with [U-^13^C]-d-Glc, the parasites were harvested and soluble-form VSG (sVSG) was isolated, as described in *Experimental procedures*. The purified sVSG preparations (which, for this study, can be considered as terminal Man metabolites) were subjected to acid hydrolysis and the released sugars were converted to their trimethylsilyl (TMS) derivatives and analysed by gas chromatography-mass spectrometry (GC-MS). The electron impact spectra of the Man and Gal derivatives allow quantification of the percentages of each sugar that have come from [U-^13^C]-d-Glc and representative extracted ion chromatograms are shown in Fig. S4. The results of triplicate analyses (Fig. 8) show that essentially all of the Man and Gal were labelled with ^13^C in the absence of Man in the medium, indicating that the synthesis of all GDP-Man and UDP-Gal was via their respective *de novo* pathways involving the transformation of [U-^13^C]-d-Glc into those sugars. However, in the presence of physiological concentrations (55 µM) of unlabelled Man about 20% of the Man isolated from sVSG was now unlabelled, whereas the Gal labelling was unaffected. These data show that the relative contributions of the *de novo* and salvage pathways to GDP-Man synthesis are about 80% and 20%, respectively, when the parasites are grown at physiologically relevant concentrations of Glc and Man. However, the proportion of GDP-Man synthesis via the salvage pathway can be further raised to 90% by increasing the extracellular concentration of Man to 1.5 mM, indicating that flux through the salvage pathway can be very significant. Indeed, at 1.5 mM Man in the medium we also observed effects on Gal labelling, such that ^13^C-labelling of Gal from [U-^13^C]-d-Glc was reduced to 80%. This suggests that high (1.5 mM) concentrations of Man in the medium can drive the intracellular conversion of some Man to Glc-6P, via Man-6P and Fru-6P, and thence to UDP-Glc and UDP-Gal, even in the presence of 5 mM Glc. While it is unclear if this metabolic flexibility is ever called on under physiological conditions, it should be borne in mind when labelling trypanosomes with stable isotope- or radioactively labelled Man (particularly if glucose-free or low-glucose medium is used) as it indicates that Man-label can be introduced into a wide range of non-Man containing metabolites via conversion to Glc. This can only be obviated by using [2-^3^H]Man, where conversion of Man-6P to Fru-6P by TbPMI via the *cis*-endiol intermediate will eliminate the tritium label from C2 of Man.

In conclusion, we have demonstrated that Man metabolism and cell growth in bloodstream-form *T. brucei* can be sustained equally well either through exclusive use of the *de novo* pathway of GDP-Man synthesis or through the salvage pathway of GDP-Man synthesis or a combination of both. The ability of the salvage pathway to compensate for the *de novo* pathway effectively removes TbPMI from the list of potential drug targets for Human African Trypanosomiasis. The redundancy between the two pathways is quite surprising since the flux through the GDP-Man pathway to sustain VSG and other glycoprotein synthesis is high (> 2.5 nmol Man per 10^7^ cells per division) and the steady-state concentration and *t*_1/2_ for turnover of GDP-Man are just 40 µM and 40 s respectively (Turnock and Ferguson, 2007). Thus, given this low pool size and high turnover rate, one might have expected the *de novo* pathway to have been essential for either growth or its knockout to have caused significant growth retardation, but this does not appear to be the case. Finally, in the course of this work, we have established a sugar-free medium capable of supporting continuous parasite growth and steady-state labelling when supplemented with 5 mM [U-^13^C]-d-Glc. This is likely to have value in several metabolic studies and, in the context of this study, we have used it to show that relative flux through the *de novo* and salvage pathways to GDP-Man under physiological conditions is in the ratio of 4:1 and that, under high (1.5 mM) Man concentrations, this ratio can be dramatically shifted to 1:9 with additional conversion of Man to Glc and Gal.

## Experimental procedures

### Cloning and expression of the *TbPMI* gene

The *TbPMI* ORF was amplified from genomic DNA (gDNA) by polymerase chain reaction (PCR) using 5′-cgcGGATCCatgtcgaagttaattaaattgga-3′ forward and 5′-ataagaatGCGGCCGCtcacatatgcgatgccgaat-3′ reverse primers. After digestion with BamHI and NotI (capital letters), the PCR product was cloned into the pGEX-6P-1 (GE Healthcare) expression vector, to form a construct encoding glutathione *S*-transferase (GST) linked to the N-terminus of TbPMI via a Precision protease site. *E. coli* BL21(DE3) (Novagen) cells were transformed with the plasmid and cultures were grown at 37°C in lysogeny broth (LB) media containing ampicillin to a density of 0.5–0.7 OD_600_ units. Protein expression was induced with 0.5 mM IPTG overnight at 18°C. The cells were harvested, resuspended in lysis buffer (50 mM Tris-HCl pH 7.3, 150 mM NaCl, 1 mg ml^−1^ lysozyme) supplemented with DNase and EDTA-free protease inhibitor cocktail (Roche), and lysed in a French press. The lysate was cleared by centrifugation (20 000 *g*, 30 min, 4°C), and the supernatant was incubated with washed Sepharose-Glutathione Fast Flow beads (GE Healthcare) for 3 h at 4°C. The beads were washed extensively with buffer A (50 mM Tris-HCl pH 7.3, 150 mM NaCl). The recombinant protein TbPMI was cleaved from the beads using Precision protease, incubated overnight at 4°C. Following centrifugation (500 *g*, 5 min, 4°C), the supernatant was concentrated using Vivaspin 10 000 MWCO centrifugal concentrator (Sartorius Stedim). Further purification of TbPMI was performed using a Superose^TM^12 10/300 column equilibrated with buffer A. The main protein peak fractions were checked by SDS-PAGE and matrix-assisted laser desorption ionization-time-of-flight (MALDI-TOF) mass spectrometry. The expected and measured molecular weight of the cleaved protein TbPMI is 46.75 kDa. The TbPMI peak fractions were pooled, adjusted to 30% glycerol at a final concentration of 1.6 mg ml^−1^ TbPMI and stored in aliquots at −80°C.

### Analytical ultracentrifugation

Recombinant TbPMI was analysed by sedimentation velocity using a Beckman Optima XL-1 Analytical Ultracentrifuge with an AN50-Ti rotor at 32 000 r.p.m. at 20°C. Absorbance data (72 scans at 280 nm) were collected and analysed using the sedfit program (Schuck, 2004). TbPMI was assumed to be globular, and its density was predicted from its amino acid composition.

### Enzyme assay

The reaction mixture (100 µl of 2 mM bis-tris propane pH 7.3, 2.5 mM MgCl_2_, containing 0.1 µg of TbPMI and 0.25 mM sugar-6P) was incubated for 15 min at 37°C. The reaction was stopped with five volumes of 0.1 M NaOH and 120 µl was analysed by high-pH anion exchange chromatography (HPAEC) using a Dionex PA-100 column (250 × 2 mm) and a pulsed amperiometric detector (Dionex Corporation). The chromatography conditions started at 50 mM sodium acetate in 0.1 M NaOH, increasing to 250 mM sodium acetate in 0.1 M NaOH over 5 min, followed by an isocratic step for 10 min, with a constant flow of 0.25 ml min^−1^. Between different samples, the column was washed with 1 M sodium acetate in 0.1 M NaOH and the column pre-equilibrated with 50 mM sodium acetate in 0.1 M NaOH. Man-6P or Fru-6P (Sigma) were used as substrates for the general activity assays. To determine the pH optimum, buffers were altered from bis-tris propane buffer (pH 6.3–9.1), to sodium acetate buffer (pH 5.0 and 5.5) and CAPS buffer (pH 10.0).

### Creation of a *T. brucei* bloodstream-form *TbPMI* conditional null mutant

The upstream and downstream UTR sequences immediately adjacent to the start and stop codons of the *TbPMI* ORF were PCR-amplified using genomic DNA template and 5′-ataagaatGCGGCCGCgtaattacccaattatttgcactc-3′ and 5′-*gtttaaac*ttacggaccgtc*AAGCTT*gtcggattcctttaaactacctag-3′ and 5′-gacggtccgtaa*gtttaaacGGATCC*gactcggtgtgttggtgc-3′ and 5′-ataagtaaGCGGCCGCtaatgaagaatatttccgccagg-3′ as forward and reverse primers respectively. The two resulting PCR products were used together in a further PCR to yield a product containing the 5′-UTR linked to the 3′-UTR by a short HindIII, PmeI and BamHI cloning site (italics small and capital letters) and NotI restriction sites at each end (capital letters). The PCR product was cloned into the NotI site of pGEM-5Zf(+) vector (Promega) and *HYG* and *PAC* drug resistance genes were cloned into HindIII and BamHI restriction sites (italics capital letters) (Guther *et al*., 2001).

For the ectopic copy, the *TbPMI* ORF was amplified from the genomic clone using 5′-ccc*AAGCTT*atgtcgaagttaattaaattggattgtg-3′ and 5′-cgc*GGATCC*tcacatatgcgatgccgaatgg-3′ as forward and reverse primers respectively. The PCR product was ligated into the HindIII/BamHI cloning sites (italics capital letters) of the pLew100 tetracycline-inducible expression vector.

The described plasmids were purified using Qiagen Mini-Prep kits, linearized with NotI, precipitated and washed twice with 70% ethanol, redissolved in sterile water and used for parasite electroporation.

### Conventional cell culture

Bloodstream from *T. brucei* cells (strain 427, variant 221) were cultured in HMI-9T medium (Hirumi and Hirumi, 1989; Greig *et al*., 2009) with 10% fetal calf serum in an incubator at 37°C and 5% CO_2_. The bloodstream-form *TbPMI* conditional null mutant derived from the same cell line was grown in the presence of 0.5 µg ml^−1^ tetracycline to induce the expression of the ectopic *TbPMI* gene (permissive conditions). To cause TbPMI starvation, the cells were washed three times in tetracycline-free HMI-9T medium prior culturing in tetracycline-free medium (non-permissive conditions). Cells were counted daily and cultures were split when densities approached ∼ 3.5 × 10^6^ cells per ml. To investigate the effects of Man in the medium, cells were cultured under non-permissive conditions in a range of Man concentrations from 100 nM to 12 mM and the growth of the cultures were assessed after 3 days using the Alamar blue assay to measure the number of viable cells (Raz *et al*., 1997). The normal physiological range of free Man in human plasma is 45–65 µM (Alton *et al*., 1997).

### Modified cell culture for [U-^13^C]-d -glucose labelling under physiological conditions

HMI-9T medium (Hirumi and Hirumi, 1989; Greig *et al*., 2009) is routinely used to support the growth of bloodstream-form *T. brucei*. It is a modified version of Iscove's modified Dulbecco's minimal essential medium (IMDM) and contains 25 mM Glc. For experiments described in this article, we wanted to develop a medium that supports the growth of *T. brucei* Lister 427 bloodstream-form cells as well as HMI-9T but that contains a more physiologically relevant concentration of Glc, closer to that found in normal human plasma (i.e. 5 mM). We started with Dulbecco's modified Eagle's medium D5030 (DMEM, Sigma), which lacks Glc, and supplemented it with salts (KNO_3_, NaHCO_3_), amino acids (l-arginine, l-cystine, l-alanine, l-asparagine, l-aspartic Acid, l-glutamic Acid, l-proline), vitamins [d(+)-biotin, Vitamin B12], HEPES, adenosine hemisulphate and Phenol Red to the concentrations present in IMDM. This modified DMEM D5030 was then supplemented, in the following order, with 1 mM sodium pyruvate (Sigma), 0.16 mM thymidine (Sigma), 0.05 mM bathocuproinedisulphonic acid disodium salt (Sigma), 1 mM hypoxanthine (Sigma), 1.5 mM l-cysteine (Sigma) and 0.056 mM 1-thioglycerol (Sigma). The medium was adjusted to pH 7.3 at room temperature and filter sterilized using a 0.2 micron filter. Following filtration the medium has a pH of 7.4 at room temperature. Each litre of sterile medium was further supplemented with 1000 U of Penicillin, 1 mg of Streptomycin, 2.5 mg of G418 (Invitrogen), 4 mg of folic acid (Sigma), 7 mg of haemin (Sigma) added from a sterile-filtered 10 mg ml^−1^ stock in 50 mM NaOH, 20% (v/v) heat-inactivated dialysed fetal bovine serum (PAA laboratories) and 20% dialysed Serum Plus (SAFC Biosciences). The Serum Plus was dialysed in snakeskin dialysis tube (Thermo Scientific, 3500 MWCO) that had been autoclaved in phosphate-buffered saline (PBS). Aliquots of 100 ml of Serum Plus were dialysed three times against 1 l of PBS for 24 h at 4°C under sterile conditions. This modified DMEM D5030 medium was assayed for Glc content using Glucose (GO) Assay Kit (Sigma) and shown to contain < 15 µM Glc. Supplementation of the media with Man (Sigma), Glc (Sigma) or [U-^13^C]-d-Glc (Cambridge Isotope Laboratories) was from sterile filtered stock solutions. Supplemented with 5 mM Glc, the modified DMDM D5030 inoculated with 5000 cells ml^−1^ supported more than eight cell doublings over 3 days, allowing steady-state labelling of these cells with [U-^13^C]-d-Glc.

### Southern blotting

*Trypanosoma brucei* genomic DNA was extracted from 100 ml of cell culture with a density of 2.5–2.8 × 10^6^ cells ml^−1^ using DNAzol (Helena Biosciences) and ethanol precipitation. Aliquots of ∼ 8 µg of gDNA were digested with various appropriate restriction enzymes overnight at 37°C, separated by agarose gel electrophoresis and blotted onto a positively charged nylon membrane (Roche). The Southern blots were UV cross-linked and hybridized with a digoxigenin-dUTP (DIG) labelled probe (Roche) that had been made by PCR using *TbPMI* ORF as template. The membranes were washed and developed with the DIG kit (Roche) and exposed to a chemiluminescence film (Amersham).

### TbPMI localization by immunofluorescence and Western blotting

Two BALB/c adult mice were used to raise polyclonal antibodies against untagged TbPMI. About 0.1 mg per mouse was used for immunization with Freund's complete adjuvant and two further 0.05 mg immunizations with Freund's incomplete adjuvant over 2 months. Immunofluorescence was performed using wild-type bloodstream-form *T. brucei* cells grown in HMI-9T medium to a density of 1 × 10^6^ cells ml^−1^, harvested by centrifugation and resuspended in trypanosome dilution buffer (20 mM Na_2_HPO_4_, 2 mM NaH_2_PO_4_, 5 mM KCl, 80 mM NaCl, 1 mM MgSO_4_, 20 mM Glc pH 7.8) to a density of 2 × 10^7^ cells ml^−1^. Aliquots (15 µl) were added to 13 mm coverslips, left at 4°C for 15 min, fixed in 1 ml of 4% paraformaldehyde in PBS for 30 min followed by three washes in 2 ml of PBS. Cells were permeabilized with 0.1% Triton X-100 in PBS for 10 min at room temperature. Samples were then blocked in 5% fish skin gelatin (FSG) in PBS containing 10% normal goat serum. The coverslips were incubated for 1 h with 1:1000 dilution of mouse anti-TbPMI antiserum and 1:4000 dilution of rabbit anti-glyceraldehyde-3-phosphate dehydrogenase (GAPDH) antiserum or 1:5000 dilution of rabbit anti-enolase antiserum in 1% FSG in PBS, 0.05% TX-100. Both anti-GAPDH and anti-enolase were kind gifts from Paul Michels (Catholic University of Louvain, Belgium). Samples were then washed in 1% FSG in PBS, 0.05% TX-100, and incubated with 50 µl of 1:500 dilution of Alexa-fluor 488-conjugated goat anti-mouse IgG and Alexa-fluor 594-conjugated goat anti-rabbit IgG for 1 h. Coverslips were washed and mounted on glass slides, sealed with Hydromount containing 2.5% DABCO and left to set in the dark and sealed with nail varnish. Microscopy was performed on a Zeiss LSM 510 META confocal microscope.

Bloodstream-form cells (5 × 10^6^) were lysed in SDS-sample buffer, 0.1 M dithiothreitol and run on a 4–12% Nupage gel with MOPS running buffer (Invitrogen), transferred to nitrocellulose and developed using 1:1000 mouse anti-TbPMI, 1:100 000 anti-mouse horseradish peroxidase conjugate (Stratech, UK) and West Pico ECL (Pierce/ThermoScientific) and Hyperfilm ECL film (GE Healthcare, UK).

### Purification of sVSG

The VSG coat of trypanosomes can be conveniently released in a soluble form (sVSG) through osmotic cell lysis at 37°C (Ferguson *et al*., 1986). We used a modified version of this procedure, also previously described (Manthri *et al*., 2008). Briefly, *T. brucei* cultures (100 ml) were washed in trypanosome dilution buffer, resuspended in 300 µl of lysis buffer (10 mM NaH_2_PO_4_-Na_2_HPO_4_, pH 8.0, 0.1 mM TLCK, 1 µg ml^−1^ leupeptin and 1 µg ml^−1^ aprotinin), and incubated at 37°C for 10 min. The lysate was then cooled on ice for 2 min and centrifuged at 16 000 *g* for 5 min. The supernatant was applied to 200 µl of DE52 (Whatman) pre-equilibrated in 10 mM NaH_2_PO_4_-Na_2_HPO_4_, pH 8.0, buffer and eluted four times with 200 µl of fresh lysis buffer. The eluates were pooled and concentrated to 100 µl using a YM-10 spin concentrator (Microcon), yielding about 50 µg of sVSG. The majority of the buffer salts were removed by diafiltration with three additions of 0.5 ml of water. This procedure yields sVSG preparations that are sufficiently pure for analysis by mass spectrometry (Manthri *et al*., 2008; Marino *et al*., 2011).

### Monosaccharide analysis of sVSG by GC-MS

Aliquots of 10 µg of sVSG were transferred to glass capillary tubes (Drummond Scientific, Broomall, PA, 100–200 µl) that had been heat-cleaned (500°C for 3 h) and flame-sealed at one end to create a microtube. For each batch of samples a set of standards of 1 nmol of Man, Gal and Glc were also placed in microtubes. Each sample was mixed with 1 nmol of *scyllo*-inositol internal standard and dried in a SpeedVac. Twenty microlitres of methanol was added and samples dried again. Then 50 µl of 4 M trifluoroacetic acid (THERMO Scientific, Rockwell, USA) was added to each tube, which after flame-sealing under a slight vacuum, were incubated at 100°C for 4 h in a heating block. After cooling, the tubes were scored with a glass knife and broken open and the samples dried in a SpeedVac. Twenty-five microlitres of MilliQ water was added to each sample, samples were re-dried, and then 25 µl of methanol was added and samples were dried once more. Finally, 15 µl of fresh TMS reagent [l-trimethylchlorosilane/hexamethyldisilazane/dry pyridine (1:3:10, v/v/v)] was added and the tubes sealed with Teflon tape. After 20–30 min at room temperature, 1 µl aliquots of the resulting hexose-TMS_6_ derivatives were then analysed by GC-MS using selected ion monitoring for the characteristic *m/z* 204 fragment ion of hexose-TMS_6_ and the corresponding *m/z* 206 ion for the ^13^C-labelled hexose. GC-MS was performed with a Hewlett-Packard 6890-5973 system. Splitless injection (injection temperature 280°C) onto a 30 m × 0.25 mm HP5 (Agilent, Wilmington, DE) column was used, using helium as the carrier gas. The initial oven temperature was 80°C (2 min), followed by temperature gradients to 140°C at 30°C min^−1^, from 140°C to 250°C at 5°C min^−1^, and from 250°C to 265°C at 15°C min^−1^. The final temperature was held for 10 min. Quantification was made by area under the curve.
