# Supplementary material for: The de novo and salvage pathways of GDP-mannose biosynthesis are both sufficient for the growth of bloodstream-form Trypanosoma brucei
Source: Mol Microbiol. 2012 Apr;84(2):340–51. doi: 10.1111/j.1365-2958.2012.08026.x (PMC3412276; doi:10.1111/j.1365-2958.2012.08026.x)
Supplement: Supplementary file 1 [file mmi0084-0340-SD1.pdf]

Supporting Information to

The *de novo* and salvage pathways of GDP-mannose biosynthesis are both sufficient for the growth of bloodstream form *Trypanosoma brucei*

Sabine Kuettel, Majken C.T. Wadum, Maria Lucia S. Güther,

Karina Mariño[1], Carolin Riemer[2], Michael A. J. Ferguson*

Division of Biological Chemistry and Drug Discovery, School of Life Sciences,

University of Dundee, Dundee DD1 5EH, Scotland, United Kingdom

*Corresponding author. Mailing address: Division of Biological Chemistry and Drug Discovery, School of Life Sciences, University of Dundee, Dundee DD1 5EH, Scotland, United Kingdom. Phone: 44 1382 384219. Fax: 44 1382 348896. E-mail: m.a.j.ferguson@dundee.ac.uk

Supplementary figures:

Figure legends

Fig. S1. Sequence alignment of PMI shows the conserved amino acids between the different organisms. The *pmi* sequence of *Trypanosoma brucei* is compared with other protozoans *Trypanosoma cruzi* and *Leishmania donovani*, with yeasts (*Saccharomyces cerevisiae* and *Candida albicans*) and with mammals (*Homo sapiens*).

Fig. S2. RT-PCR. *TbPMI* is transcribed in both the procyclic and bloodstream form life cycle stages of the *T.brucei*. The RT-PCR was performed using TbPMI primers, DPMS (Dolichyl-phosphate-mannose synthetase) primers as a positive control and DPMS primers without reverse transcriptase as a negative control.
(A) RT-PCR product of procyclic RNA, used in different amounts. (B) RT-PCR product of bloodstream RNA, used in different amounts. (1) Procyclic negative control. (2) Procyclic positive control. (3) Bloodstream negative control. (4) Bloodstream positive control.

Fig. S3. The optimum pH for TbPMI activity was determinded by performing reactions over a range of pH values from pH 5.0 to pH 10. The reactions were performed in triplicate for each pH value. TbPMI was found to have a pH optimum between pH 7.3 and 8.5.

Fig. S4. Gas chromatography-mass spectrometry (GC-MS). Monosaccharides released from VSG-glycoconjugates by acid hydrolysis (4 M TFA, 100°C, 4 hours) were TMS-derivatized and then analyzed by GC-MS. The ions 204 and 206 corresponding to ^{12}C- and ^{13}C-hexoses, respectively, were extracted from the GC-MS total ion chromatogram of acid hydrolyzed, TMS-derivatized (A) mannose and galactose standards (1 nmol each) or VSG (10 μg) isolated from wild type *T.brucei* bloodstream cells grown in (B) 5 mM ^{13}C-glucose, (C) 5 mM ^{13}C-glucose and 55 μM ^{12}C-mannose, or (D) 5 mM ^{13}C-glucose and 1.5 mM ^{12}C-mannose.

Fig. S1:

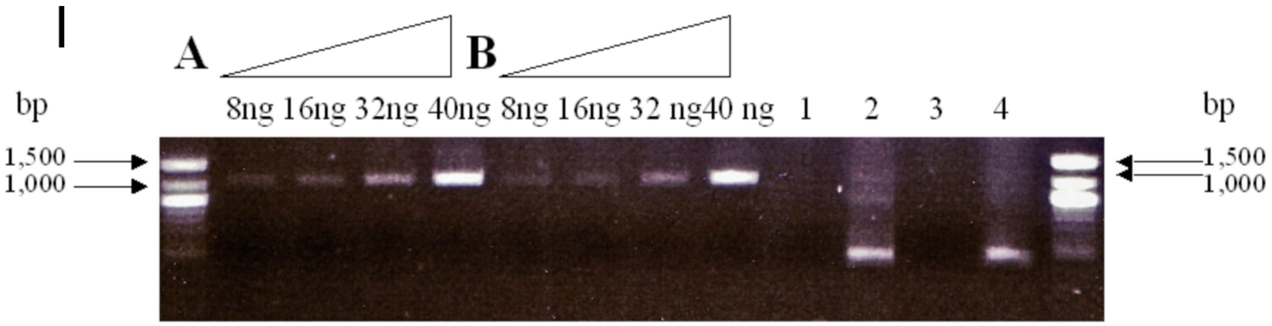
A
B
bp
8ng 16ng 32ng 40ng 8ng 16ng 32 ng40 ng 1 2 3 4
bp
1,500
1,000
1,500
1,000

Fig. S2:

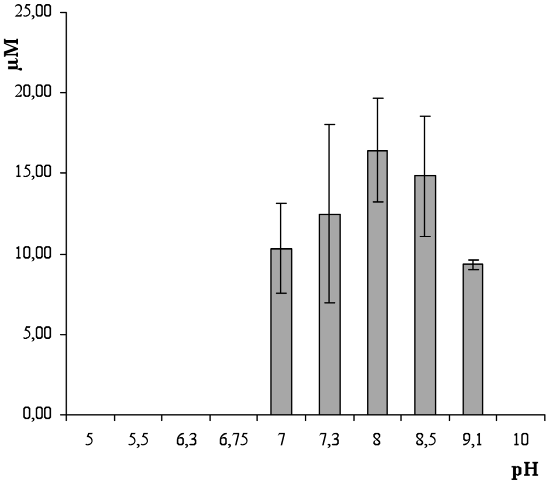

Fig. S3:

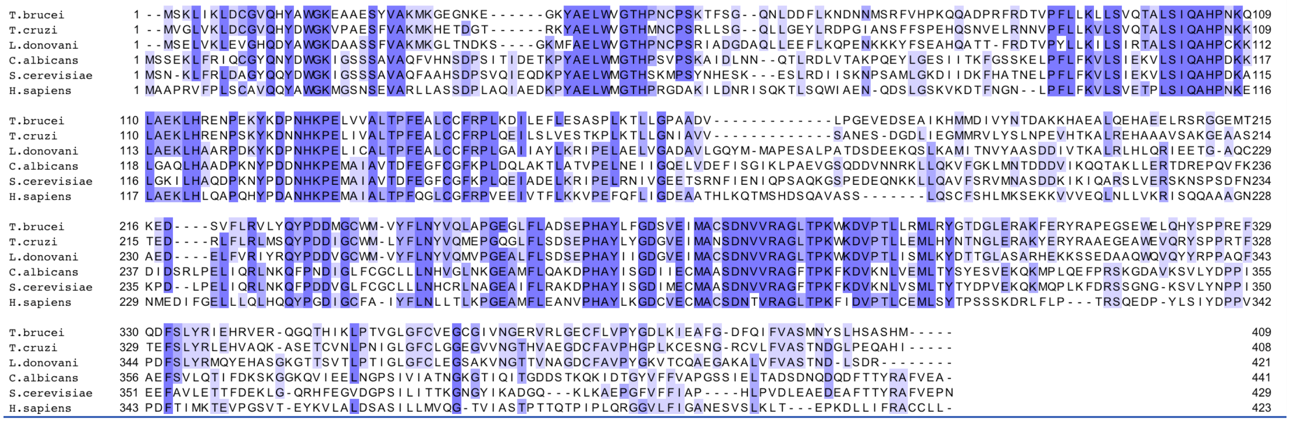

Fig. S4:

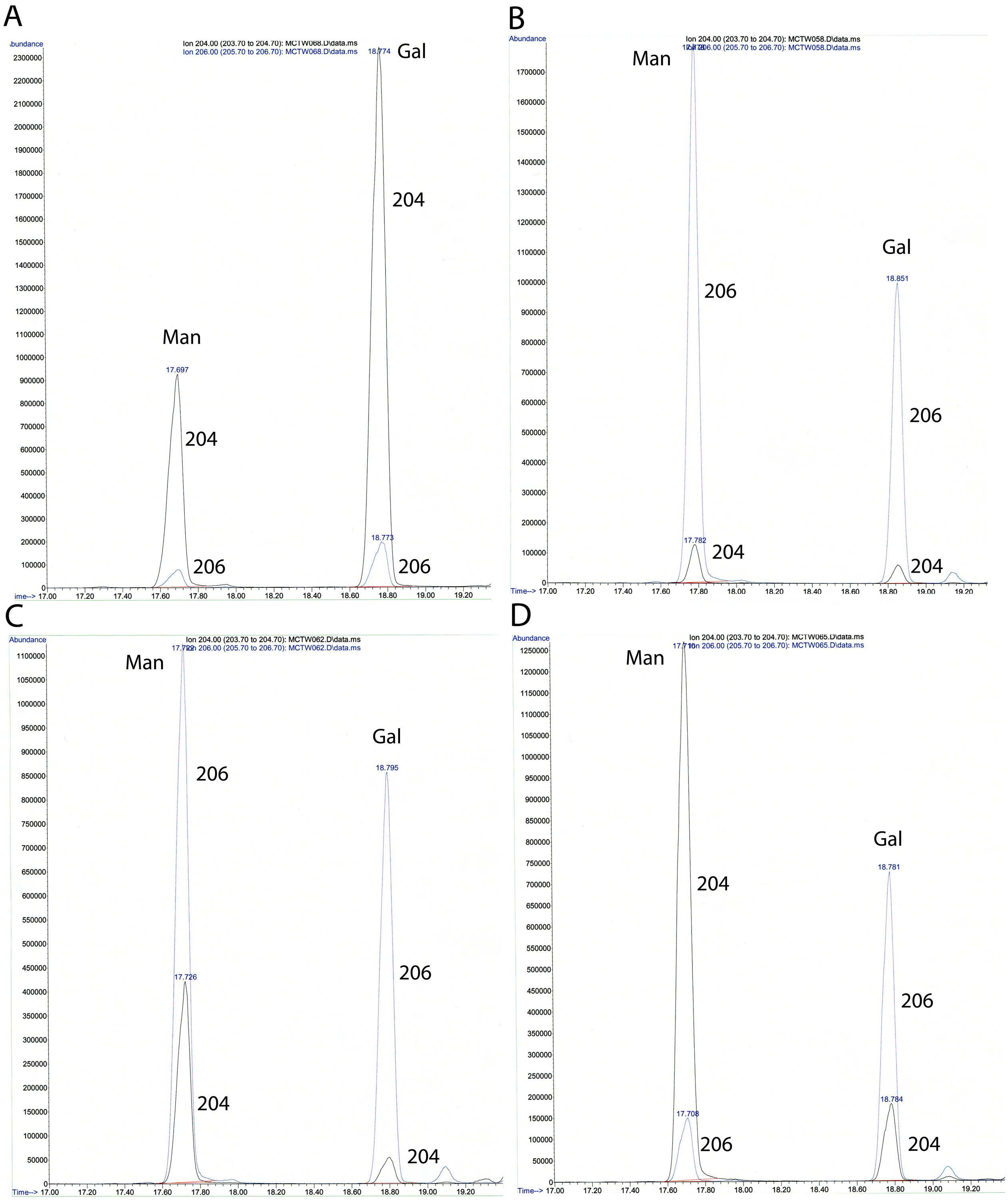
A
Abundance
Ion 204.00 (203.70 to 204.70): MCTW068.D\data.ms
Ion 206.00 (205.70 to 206.70): MCTW068.D\data.ms
Man
17.697
204
206
Gal
18.774
204
18.773
206
B
Abundance
Ion 204.00 (203.70 to 204.70): MCTW058.D\data.ms
Ion 206.00 (205.70 to 206.70): MCTW058.D\data.ms
Man
206
17.782
204
Gal
18.851
206
204
C
Abundance
Ion 204.00 (203.70 to 204.70): MCTW062.D\data.ms
Ion 206.00 (205.70 to 206.70): MCTW062.D\data.ms
Man
206
17.726
204
Gal
18.795
206
204
D
Abundance
Ion 204.00 (203.70 to 204.70): MCTW065.D\data.ms
Ion 206.00 (205.70 to 206.70): MCTW065.D\data.ms
Man
204
17.708
206
Gal
18.781
206
18.784
204
Time-->
